# Clinical study of cardiac autonomic dysfunction in HIV patients

**DOI:** 10.1186/1471-2334-14-S3-P35

**Published:** 2014-05-27

**Authors:** Pradheep C Mathew, Shishir Nagesh Duble

**Affiliations:** 1 Department of General Medicine, Rajiv Gandhi University, AJ Institute of Medical Sciences, Mangalore, Karnataka, India

## Background

Cardiovascular autonomic nervous dysfunction has been demonstrated to severely debilitate HIV infected patients, namely by postural hypotension and syncopes. It has important implication in health care of HIV patients. Presence of autonomic neuropathy signals the need for added precautions when invasive procedures are performed on HIV patients.

## Methods

Fifty patients (25 HIV +ve without AIDS and 25 HIV+ with AIDS) who fulfilled the inclusion and exclusion criteria and 50 healthy matched controls were enrolled in the study. All HIV positive/AIDS patients were evaluated according to a detailed proforma with elicitation of history, symptoms, signs and routine and specialized investigations.

## Results

In the present study, 16% of HIV +ve with the AIDS had abnormal autonomic dysfunction and 4% of HIV positive without AIDS had abnormal autonomic dysfunction. Reduced heart rate variability is the commonest manifestation of autonomic dysfunction noted in both HIV positive without AIDS and HIV positive with AIDS groups. Diastolic BP response to sustained handgrip has a limited role in discriminating autonomic function in HIV infected patients. There is no statistically significant correlation with the CD4 level and the presence of autonomic nervous system dysfunction in both the groups.

## Conclusion

Cardiac autonomic nervous dysfunction is a common and relevant clinical problem affecting both HIV positive without AIDS and HIV positive with AIDS groups. It may provide an alternative explanation for symptoms commonly observed in HIV infected individuals such as bowel and bladder dysfunction, impotence, syncope and sweating abnormalities.

